# Interaction Between Patient-Related and Surgical Factors in the Pattern of Voice Recovery Following Thyroidectomy: A Prospective Cohort Study

**DOI:** 10.3390/diagnostics16182931

**Published:** 2026-09-10

**Authors:** Ivana Šimić Prgomet, Jakov Bilać, Boris Bumber

**Affiliations:** 1 Phoniatric Center, Department of ENT and Head and Neck Surgery, University Hospital Center Zagreb, Kišpatićeva 12, 10000 Zagreb, Croatia; simic.ivana24@gmail.com; 2 Department of ENT and Head and Neck Surgery, University Hospital Center Zagreb, Kišpatićeva 12, 10000 Zagreb, Croatia; borisbumber@yahoo.com; 3 Department of ENT, General Hospital Šibenik, Ulica Stjepana Radića 83, 22000 Šibenik, Croatia

**Keywords:** thyroidectomy, objective voice outcomes, postoperative voice recovery, body mass index, surgery duration, recurrent laryngeal nerve

## Abstract

**Background:** A substantial proportion of patients experience persistent voice-related symptoms after thyroidectomy, even without clinically detectable laryngeal nerve injury. Identifying factors associated with delayed voice recovery is therefore important, as postoperative voice alterations may influence vocal performance and occupational voice use. This study examined the associations of patient-related (age, sex, body mass index [BMI]) and surgical variables (thyroid volume, type, and duration of surgery) with postoperative voice recovery stage within a six-month follow-up period. **Methods**: This prospective cohort study included 292 consecutive adults undergoing thyroidectomy at a tertiary referral center with normal preoperative voice and laryngeal findings. Patients with prior neck surgery, pre-existing voice disorders, reflux disease, or intraoperative laryngeal nerve injury were excluded. Objective voice assessment was performed multidimensionally at four predefined postoperative time points. The recovery stage was defined ordinally according to the first follow-up demonstrating normalization of all objective voice parameters. Multinomial logistic regression was used to examine multivariable associations and interactions between BMI and surgical variables. **Results**: Three regression models examined interactions between BMI and surgical variables, with recovery stage 4 (no normalization by six months) as the reference category. All three models were statistically significant (χ^2^(15) ≥ 86.32, *p* < 0.001). Duration of surgery was independently associated with recovery stage across models, with longer surgery associated with lower odds of recovery at stages 2 and 3 relative to stage 4 (*p* ≤ 0.041). Thyroid volume was also associated with recovery, with higher volume associated with greater odds of stage 1 versus stage 4 recovery (*p* = 0.008). Significant interactions were observed between BMI and duration of surgery (LR χ^2^(3) = 12.10, *p* = 0.007) and between BMI and type of surgery (LR χ^2^(3) = 15.17, *p* = 0.002), both driven by the comparison of stage 3 with stage 4. No significant interaction was found between BMI and thyroid volume (*p* = 0.294). **Conclusions**: Both patient-related (BMI) and surgical factors were associated with postoperative voice recovery stage. The association between BMI and recovery differed according to surgical duration and type, but not thyroid volume. These findings may support risk stratification and more tailored postoperative monitoring of objective postoperative voice recovery. Future multicenter studies using appropriate ordinal regression models and interval-censored survival methods are warranted to further clarify individualized recovery patterns following thyroidectomy.

## 1. Introduction

Thyroidectomy is one of the most commonly performed surgical procedures in head and neck practice. Depending on the underlying indication, the procedure may involve removal of a single thyroid lobe (lobectomy) or complete removal of the gland (total thyroidectomy) [1]. Owing to the close anatomical relationship between the thyroid gland, the laryngeal structures involved in phonation, and their associated neural supply, thyroidectomy is frequently associated with postoperative voice impairment [2].

Traditionally, postoperative voice changes have been primarily attributed to injury of the recurrent laryngeal nerve (RLN), resulting in vocal fold paresis or paralysis. With advances in surgical techniques and routine identification of the RLN, the incidence of transient nerve injury has decreased to approximately 10%, while permanent RLN injury is reported in only 0.5–2% of cases [3]. However, accumulating evidence indicates that a substantial proportion of patients experience persistent voice-related symptoms even in the absence of clinically detectable laryngeal nerve injury, as reflected by preserved vocal fold mobility [4,5]. These symptoms may include vocal fatigue, roughness, reduced vocal intensity, and difficulties with high-pitched phonation and singing, with some studies reporting persistence beyond 18 months after thyroidectomy [6].

Identifying factors associated with prolonged voice recovery after thyroidectomy is of particular importance, as persistent voice dysfunction may influence vocal performance and occupational voice use [7]. Several studies have examined risk factors for impaired voice outcomes after thyroidectomy, with inconsistent results [8,9,10,11]. Most of these studies focused on individual variables in isolation, including sociodemographics, occupational voice use, smoking status, extent and duration of surgery, and preoperative thyroid volume or function, typically using univariate analyses or simple regression models [12,13]. A smaller subset of studies has focused specifically on prolonged voice recovery, identifying predictors of delayed return to normal voice function. Reported factors include race, early changes in acoustic or perceptual voice measures, and professional voice use [14,15,16,17]. In our previous prospective cohort study, we characterized the trajectory of postoperative voice changes in patients without RLN injury and identified higher body mass index (BMI), total thyroidectomy, longer surgery duration, and larger preoperative thyroid volume as factors associated with impaired voice recovery [18].

Building on these findings, the present study aims to examine the associations of patient-related (age, sex, BMI) and surgical variables (thyroid volume, type, and duration of surgery) with postoperative voice recovery stage within a six-month follow-up period. Particular emphasis is placed on evaluating the main and interaction effects of these factors on the recovery stage assessed at predefined postoperative time points.

## 2. Materials and Methods

### 2.1. Study Design and Setting

We conducted a hospital-based prospective cohort study at the Department of Otorhinolaryngology—Head and Neck Surgery, University Hospital Centre Zagreb, from March 2023 to July 2024. This study was approved by the hospital Research Ethics Committee and was conducted and reported in accordance with the Strengthening the Reporting of Observational Studies in Epidemiology (STROBE) guidelines [19]. The purpose and procedures were explained to each participant, and informed consent was obtained prior to enrollment. The overall study protocol was based on our previously published investigation [18], with modifications described as follows.

### 2.2. Participants

Eligible participants were adults undergoing thyroidectomy at our institution for benign thyroid disease or well-differentiated thyroid carcinoma confined to the thyroid gland, with normal preoperative laryngeal endoscopy and voice assessment. The study cohort consisted exclusively of patients undergoing lobectomy or total thyroidectomy without concomitant central or lateral neck dissection. The extent of thyroid resection (lobectomy or total thyroidectomy) was determined according to standard clinical and oncological indications and was independent of the study protocol. Exclusion criteria included pre-existing voice impairment of any cause, clinical or pathological evidence of extrathyroidal extension or nodal disease requiring neck dissection, history of neck surgery, laryngopharyngeal reflux (LPR) or gastroesophageal reflux disease (GERD), and intraoperative RLN injury.

All consecutive patients meeting the criteria were enrolled, resulting in a final cohort of 292 participants. No formal sample size calculation was performed; the sample reflects all eligible patients during the study period.

### 2.3. Surgical Procedure

All surgeries were performed under general anesthesia with endotracheal intubation by a single high-volume thyroid surgeon. The procedure included extracapsular total thyroidectomy or lobectomy through a collar incision with midline opening of the fascia. Strap muscles were preserved unless necessary. Operation technique included routine identification and preservation of RLN using surgical loupes without the use of intraoperative neuromonitoring. External branch of the superior laryngeal nerve (EBSLN) was not routinely identified, but care was taken to avoid accidentally injuring it by accurate dissection close to the thyroid capsule and by using a single ligature of the superior lobe.

### 2.4. Follow-Up Schedule

Participants were evaluated at four points of measurement (POM): seven days preoperatively (POM1), first postoperative follow-up between the seventh and tenth day after surgery (POM2), second postoperative follow-up three months after surgery (POM3), and third postoperative follow-up six months after surgery (POM4).

Duration of voice recovery was operationalized as the postoperative point of measurement at which complete normalization of all voice parameters was first achieved. Recovery stage was coded as follows: 1 = recovery by POM2 (7–10 days postoperatively), 2 = recovery by POM3 (3 months), 3 = recovery by POM4 (6 months), and 4 = no normalization at POM4.

During follow-up, patients who achieved complete normalization of all voice parameters at a given postoperative assessment were classified as recovered and were not scheduled for further clinical voice evaluations. For repeated-measures analyses, recovery was treated as an absorbing state, meaning it was assumed to be sustained once normalization had been achieved. Accordingly, the last observed normalized values were carried forward to all subsequent postoperative time points, preserving the longitudinal structure of the dataset and facilitating repeated-measures analyses across all scheduled assessments. Therefore, the reduction in the number of participants undergoing direct evaluation at successive follow-up visits reflected achievement of full voice recovery rather than attrition.

### 2.5. Voice Assessment

Voice assessment was multidimensional, including perceptual assessment using the Grade, Roughness, Breathiness, Asthenia, Strain (GRBAS) scale, acoustic measures (fundamental frequency, intensity, jitter, shimmer), aerodynamic assessment (maximum phonation time), and laryngeal imaging (videostroboscopy). Patient-reported voice outcomes were also collected as part of the study protocol but were not included in the present analysis, as it was specifically focused on objective postoperative voice recovery parameters. Accordingly, all outcome definitions and recovery-stage classifications were based exclusively on objective voice measures.

Perceptual voice quality was evaluated using the GRBAS scale originally described by Hirano [20], selected because it remains the most widely used and clinically validated perceptual voice assessment tool in voice-disorder research, providing a standardized and reproducible measure of overall voice quality that facilitates comparison with prior studies. Two experienced speech-language pathologists independently rated all recordings. Interrater reliability, calculated as percentage agreement, ranged from 88% to 96% across postoperative assessments (100% preoperatively). In cases of disagreement, the final score was calculated as the mean of the two raters’ scores. Although percentage agreement provides an intuitive index of consistency, it does not correct for chance agreement; reporting a chance-corrected statistic such as weighted Cohen’s kappa or the intraclass correlation coefficient would be preferable and is recommended for future studies.

Objective voice evaluation included acoustic and aerodynamic measures performed in a sound-treated laboratory to minimize external noise. Recordings were obtained using a digital recorder and a head-mounted microphone positioned 30 cm from the participant’s mouth at a 45° angle. Acoustic analysis was conducted using LingWAVES SLP Suite Pro VPR, version 3.2 (WEVOSYS Medical Technology GmbH, Baunach, Germany). Measured parameters included fundamental frequency (F0), intensity, jitter, and shimmer during sustained vowel/a/phonation. Aerodynamic assessment included maximum phonation time (MPT) as an indicator of phonatory efficiency. Reference values were defined using fixed thresholds from published norms and the LingWAVES Voice Protocol [21]: F0 (female ~206 Hz, male ~120 Hz), intensity at habitual loudness (female 68–74 dB, male 68–76 dB), jitter (≤0.5%), shimmer (≤5%), and MPT (female 15–25 s, male 25–35 s). Full recovery at a given time point required the simultaneous normalization of all of the above parameters, defined operationally as a GRBAS score of 0 on every subscale together with F0, intensity, jitter, and shimmer within the reference ranges specified above and MPT within the corresponding normative range; patients not meeting all of these criteria concurrently were classified as not yet recovered at that time point.

Videostroboscopy was performed with the KayPENTAX Laryngeal Strobe Model 9400 (KayPENTAX, Montvale, NJ, USA). to determine voice status in preoperative assessment and postoperative evaluation of vocal fold function, including detection of vocal fold injury and impaired vocal fold mobility at the first postoperative follow-up visit.

Voice impairment was defined as deviation from reference values in any perceptual, acoustic, or aerodynamic measure. Duration of voice recovery was defined as the postoperative time point at which each patient achieved normalization of all voice parameters. Both preoperative voice status and postoperative recovery were determined according to predefined normative reference thresholds for each perceptual, acoustic, and aerodynamic parameter rather than by comparison with individual baseline values. This approach was adopted because all participants met the inclusion criterion of normal preoperative voice and laryngeal findings, allowing a common, clinically interpretable normative benchmark to be applied consistently across the cohort; nevertheless, this approach cannot capture within-subject deviations from each patient’s own preoperative values, a point that is addressed further as a limitation.

### 2.6. Covariates and Comorbidities

Sociodemographic data (age, sex, educational level, voice profession status), body mass index (BMI), type of surgery (lobectomy or total thyroidectomy), duration of surgery, preoperative thyroid volume, and comorbidities were collected using a study-specific questionnaire. BMI was categorized according to World Health Organization (WHO) criteria as underweight (<18.5 kg/m^2^), normal weight (18.5–24.9 kg/m^2^), overweight (≥25.0 kg/m^2^), and obesity (≥30.0 kg/m^2^) [22]. Thyroid volume was determined based on ultrasound measurements of the gland using sex-specific cutoff values. In males, volume above 25 mL was classified as enlarged thyroid, whereas in females, above 18 mL [23]. Duration of surgery was recorded as a continuous variable in minutes. For the purpose of group-based analyses, patients were categorized using the sample median duration of surgery (90 min) as the cutoff value, resulting in two groups (≤90 min and >90 min). Diagnosis of LPR was based on clinical history using the Reflux Symptom Index (RSI) for screening [24] and confirmed with laryngoscopic examination using the Reflux Finding Score (RFS) [25]. GERD was based on a previously documented diagnosis by a gastroenterologist. The RSI and RFS were selected because they are validated, widely used instruments for reflux-related symptom and sign screening in laryngological practice, allowing systematic and reproducible identification of patients with laryngopharyngeal reflux for exclusion from the study cohort.

### 2.7. Statistical Analysis

Continuous variables were summarized using appropriate descriptive statistics, whereas categorical variables were presented as frequencies and percentages. Associations between continuous predictors and postoperative recovery stage were initially explored using non-parametric correlation analyses, while associations involving categorical predictors were examined descriptively and using appropriate bivariate tests.

Postoperative recovery was originally coded as an ordinal outcome reflecting the timing of recovery across four postoperative assessment points. Ordinal logistic regression was initially considered because of the ordered nature of the outcome variable. However, the proportional odds assumption was not satisfied, as indicated by the Test of Parallel Lines. Therefore, multinomial logistic regression was used for the multivariable analyses.

The recovery category representing patients who had not recovered by the final postoperative assessment (stage 4) was used as the reference outcome category. Age and sex were initially considered as potential covariates; however, neither variable showed a significant bivariate association with postoperative recovery, and they were therefore not included in the final parsimonious models. This decision was based on bivariate screening rather than on a single multivariable model incorporating all six candidate predictors (age, sex, BMI, surgery type, duration of surgery, and thyroid volume) simultaneously; while excluding non-significant covariates reduces model complexity and the risk of overfitting given the available sample size, it does not entirely rule out residual confounding by age or sex, and this trade-off is acknowledged as a limitation of the analytic approach.

BMI and duration of surgery were treated as continuous variables and mean-centered before the calculation of interaction terms. Three separate multinomial logistic regression models were fitted to examine the interactions between BMI and duration of surgery, BMI and type of surgery, and BMI and thyroid volume. Each model included the main effects of BMI, duration of surgery, type of surgery, and thyroid volume, together with the respective interaction term.

Overall model significance was assessed using likelihood ratio chi-square tests. Model fit was evaluated using the Pearson goodness-of-fit test, and pseudo-R^2^ measures, including Cox and Snell, Nagelkerke, and McFadden R^2^, were reported. The contribution of individual predictors and interaction terms was evaluated using likelihood ratio tests. Results are presented as odds ratios (ORs) with 95% confidence intervals (CIs). A two-sided *p* value < 0.05 was considered statistically significant.

### 2.8. Use of Artificial Intelligence

During the preparation of this manuscript, the authors used ChatGPT (OpenAI, San Francisco, CA, USA, GPT-5.6 Luna, web-based version) solely for minor language editing, grammar checking, and improving clarity and readability. The AI tool was not used to generate scientific content, study design, data, analyses, interpretations, figures, or conclusions. The authors reviewed and edited all AI-assisted output and take full responsibility for the content of this publication.

## 3. Results

**Figure 1:**
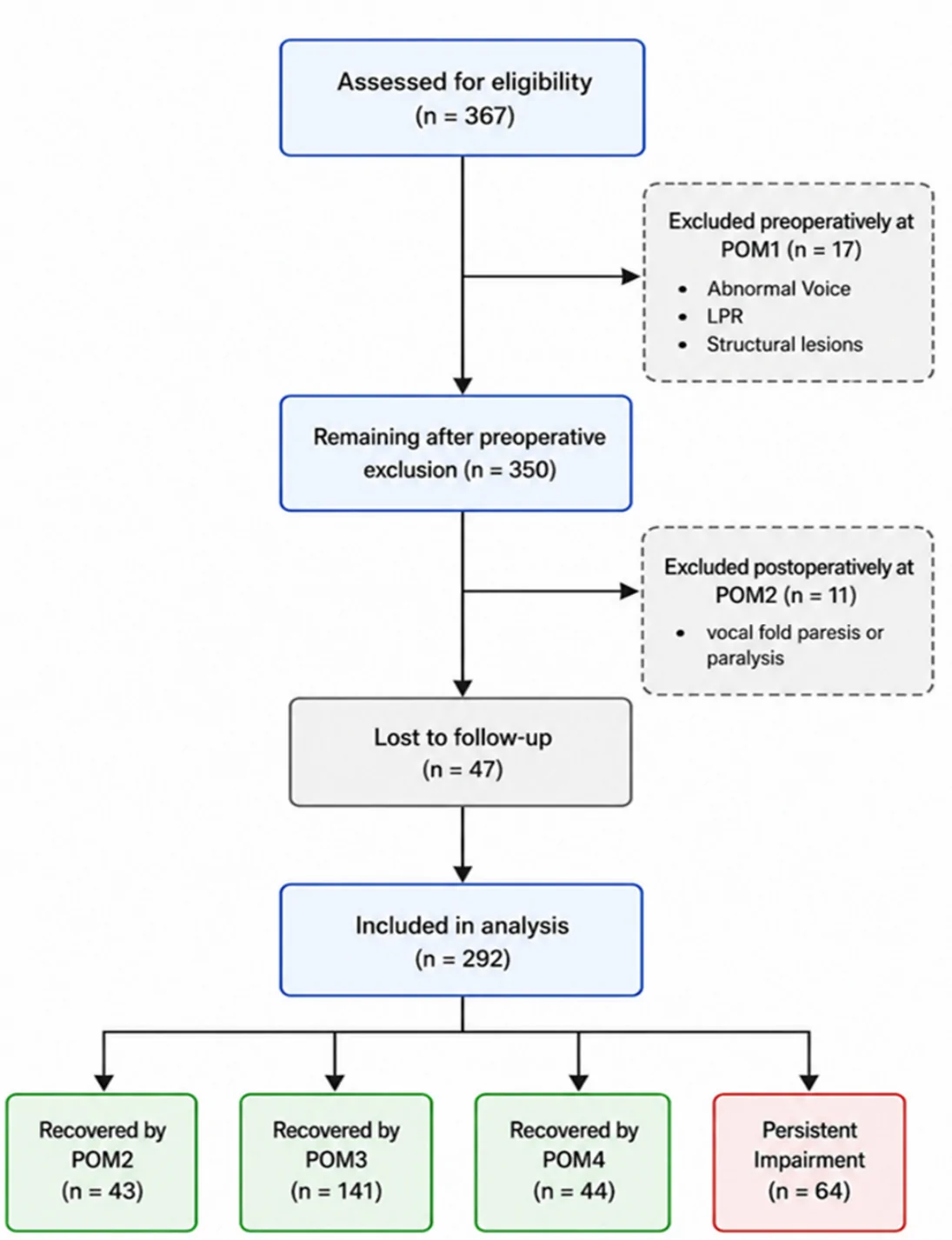
Study cohort selection and follow-up flowchart. Point of measurement (POM); laryngopharyngeal reflux (LPR).

A total of 367 patients scheduled for thyroidectomy at our institution were initially assessed for eligibility. Seventeen patients were excluded preoperatively due to abnormal baseline voice parameters or structural laryngeal findings suggestive of laryngopharyngeal reflux or vocal fold pathology. Of the remaining 350 patients, 11 were excluded after the first postoperative assessment (POM2) due to newly diagnosed vocal fold paresis or paralysis confirmed by videostroboscopy. Additionally, 47 patients were lost to follow-up at POM2. After applying all eligibility criteria, 292 patients were included in the final analysis. Among the 292 patients included in the final analysis, recovery-stage outcomes were distributed as follows: 43 patients recovered by POM2 (stage 1), 141 additional patients recovered by POM3 (stage 2), and 44 more recovered by POM4 (stage 3). At the final follow-up (POM4), 64 patients continued to exhibit non-normalized voice parameters (stage 4). A flowchart illustrating the research methodology is shown in Figure 1.

The majority of included patients were female (84.2%), with most participants aged between 40 and 59 years (56.5%). The largest number of recoveries occurred during the second recovery stage (between POM2 and POM3; *n* = 141).

Only a small proportion of patients had normal BMI; 42.8% were overweight, and 43.2% had obesity. An equal number of patients underwent lobectomy and total thyroidectomy. The duration of surgery exceeded 90 min in 46.9% of cases. Enlarged preoperative thyroid volume was observed in 53.8% of patients.

Table 1 presents the distribution of patients according to baseline characteristics, including patient-related variables (sociodemographic characteristics and BMI), surgical variables (type of surgery, duration of surgery, and preoperative thyroid volume), and postoperative recovery-stage categories.

Multinomial logistic regression analyses were performed to examine whether the association between BMI and postoperative recovery differed according to duration of surgery, type of surgery, and thyroid volume. Recovery stage 4, representing patients who had not recovered by the final postoperative assessment, was used as the reference outcome category (Table 2).

The multinomial logistic regression model examining the interaction between BMI and type of surgery was statistically significant, χ^2^(15) = 97.77, *p* < 0.001 (Table 3). The Pearson goodness-of-fit test indicated an adequate model fit (*p* = 0.738), and the Nagelkerke pseudo-R^2^ was 0.310. The interaction between BMI and type of surgery was statistically significant overall, LR χ^2^(3) = 15.17, *p* = 0.002. The interaction was statistically significant for recovery stage 3 compared with stage 4 (OR = 1.362, 95% CI [1.075, 1.724], *p* = 0.010), but not for stages 1 or 2 compared with stage 4. Thus, the association between BMI and postoperative recovery differed according to the type of surgery, particularly with respect to recovery occurring at the third postoperative assessment compared with no recovery by the final assessment. Duration of surgery remained significantly associated with recovery stage, with longer procedures associated with lower odds of recovery at stages 2 and 3 compared with stage 4.

The multinomial logistic regression model examining the interaction between BMI and duration of surgery was statistically significant, χ^2^(15) = 94.70, *p* < 0.001 (Table 4). The model showed an acceptable fit according to the Pearson goodness-of-fit test (*p* = 0.304) and explained approximately 30.2% of the variation in recovery stage according to the Nagelkerke pseudo-R^2^. The interaction between BMI and duration of surgery was statistically significant overall, LR χ^2^(3) = 12.10, *p* = 0.007. Examination of individual comparisons indicated that the interaction was statistically significant for recovery stage 3 compared with stage 4 (OR = 1.004, 95% CI [1.001, 1.007], *p* = 0.011), whereas it was not statistically significant for stages 1 or 2 compared with stage 4. These findings indicate that the association between BMI and postoperative recovery differed depending on the duration of surgery, particularly when comparing patients recovering at the third postoperative assessment with those who had not recovered by the final assessment. Duration of surgery also showed significant associations with recovery stage. Longer duration was associated with lower odds of recovery at stage 2 and stage 3 compared with remaining unrecovered at stage 4.

The multinomial logistic regression model examining the interaction between BMI and thyroid volume was statistically significant overall, χ^2^(15) = 86.32, *p* < 0.001 (Table 5). The model showed an acceptable fit according to the Pearson goodness-of-fit test (*p* = 0.372) and had a Nagelkerke pseudo-R^2^ of 0.279. However, the interaction between BMI and thyroid volume was not statistically significant, LR χ^2^(3) = 3.72, *p* = 0.294. None of the individual recovery-stage comparisons showed a statistically significant interaction effect. These findings suggest that the association between BMI and postoperative recovery did not differ significantly between patients with normal and enlarged thyroid volume. Duration of surgery remained significantly associated with recovery stage, with longer procedures associated with lower odds of recovery at stages 2 and 3 compared with patients who remained unrecovered at the final assessment.

Overall, significant interactions were observed between BMI and duration of surgery and between BMI and type of surgery, whereas no evidence was found for an interaction between BMI and thyroid volume.

### 3.1. Model 1: BMI × Type of Surgery

In Model 1, higher BMI was associated with later recovery stages among patients undergoing total thyroidectomy, but not among patients with lobectomy.

**Figure 2:**
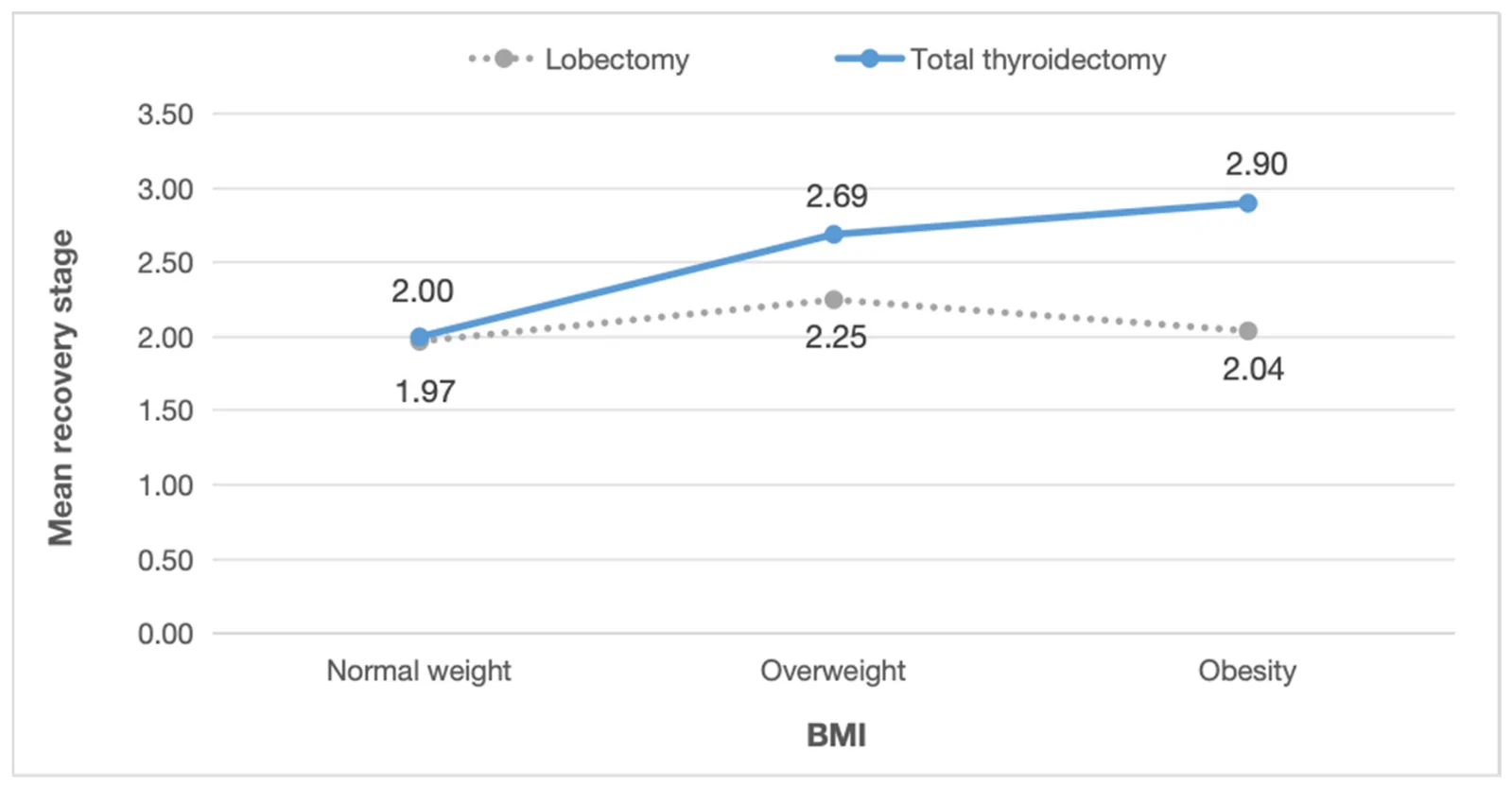
Interaction between BMI and type of surgery on recovery stage.

The pattern of main and interaction effects is illustrated in Figure 2.

### 3.2. Model 2: BMI × Duration of Surgery

In Model 2, the significant interaction indicated that, among patients with obesity, longer duration of surgery was associated with later recovery stages, whereas this association appeared less pronounced among patients with normal BMI.

**Figure 3:**
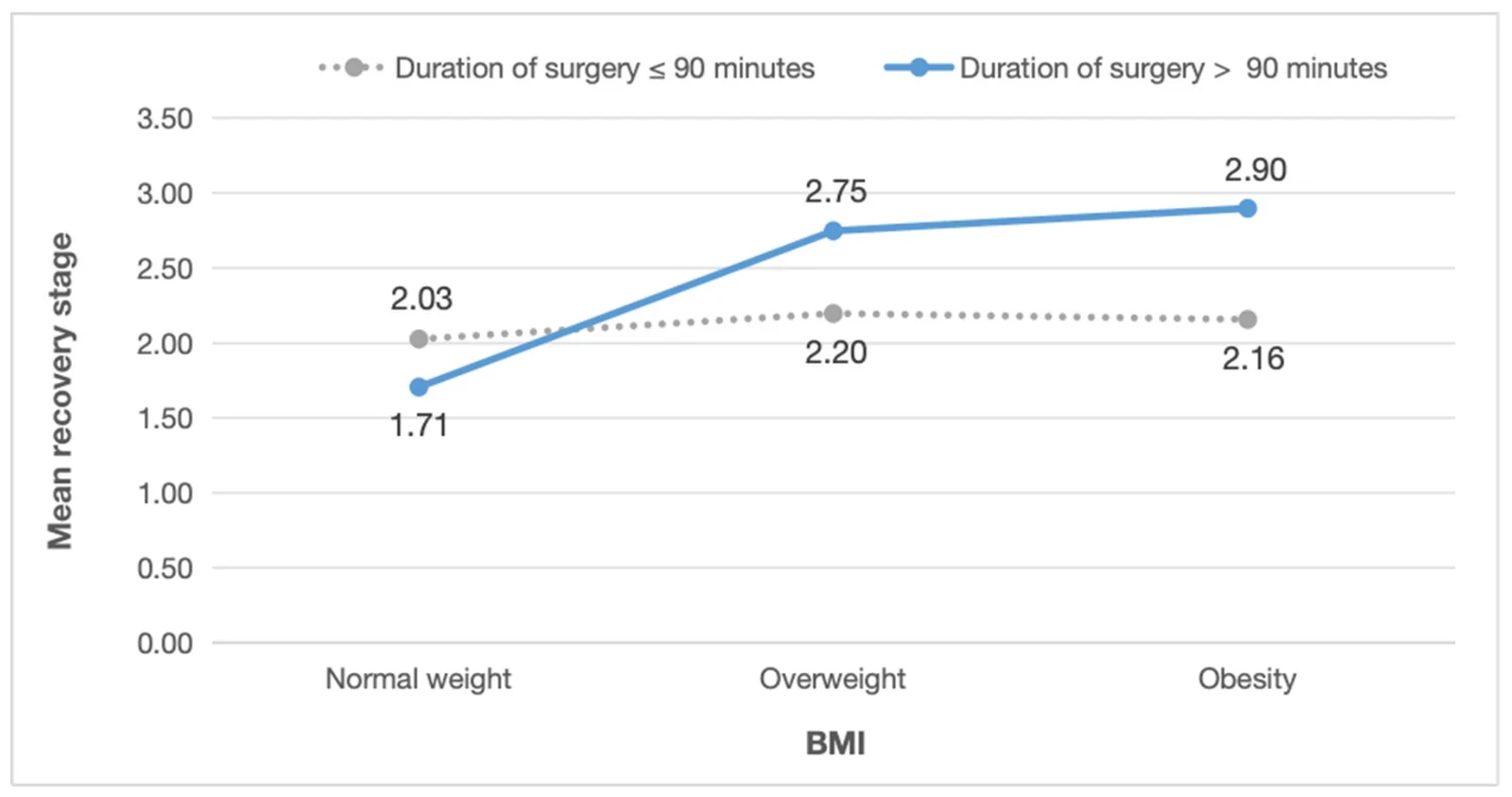
Interaction between BMI and duration of surgery on recovery stage.

The pattern of effects is illustrated in Figure 3.

### 3.3. Model 3: BMI × Preoperative Thyroid Volume

In Model 3, patients with higher BMI tended to show somewhat later recovery stages following thyroid surgery; however, the interaction between BMI and preoperative thyroid volume was not statistically significant.

**Figure 4:**
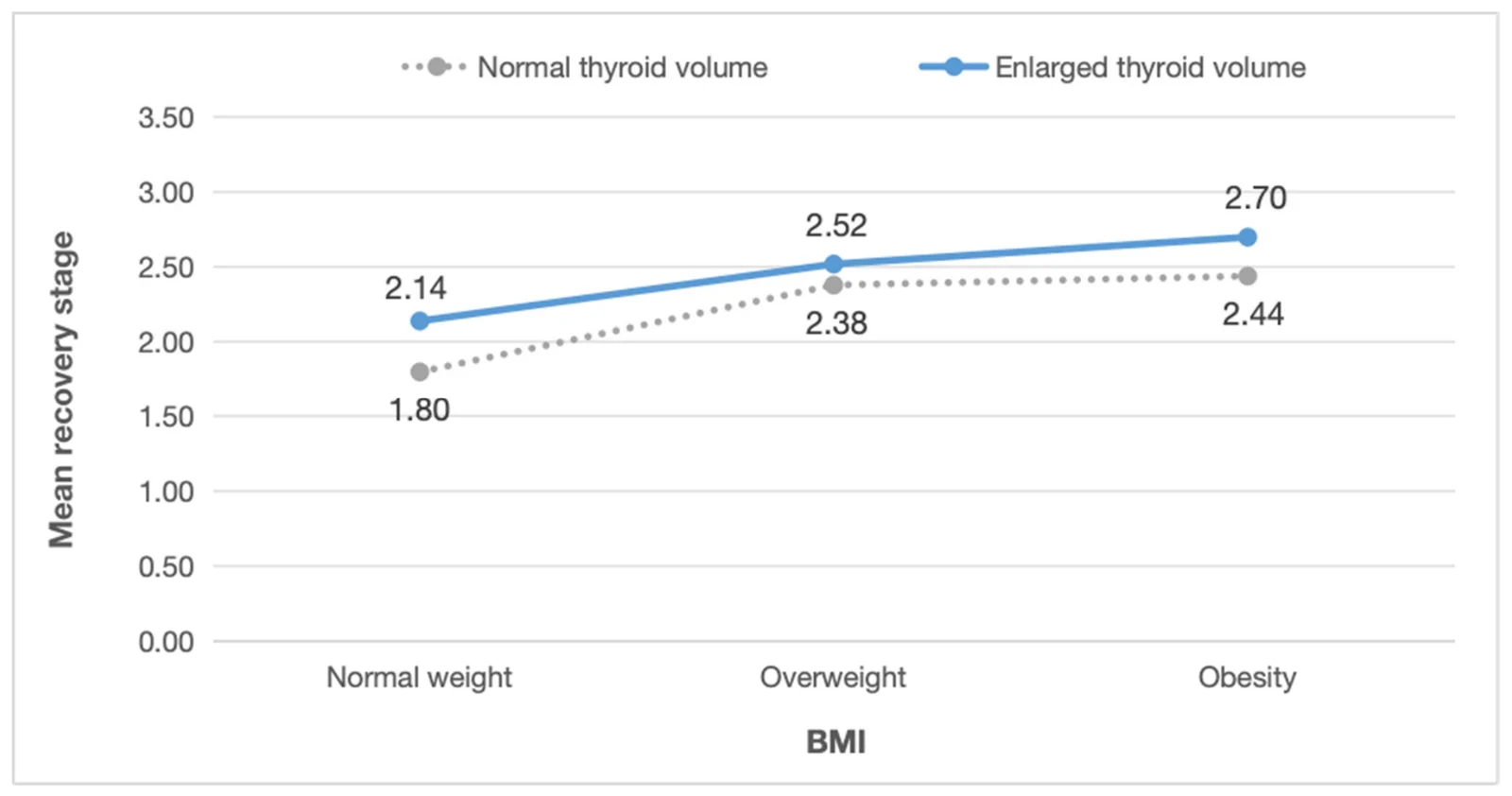
Interaction between BMI and thyroid volume on recovery stage.

The pattern of main and interaction effects is illustrated in Figure 4.

## 4. Discussion

The present study examined the associations of patient-related and surgical variables with postoperative voice recovery stage, including potential interaction effects, within a six-month follow-up period. The findings indicate that postoperative recovery stage was associated with BMI and several surgical characteristics, including type and duration of surgery and preoperative thyroid volume. Significant interaction patterns were observed between BMI and duration of surgery and between BMI and type of surgery, whereas no evidence was found for an interaction between BMI and thyroid volume. These findings suggest that the association between BMI and postoperative voice recovery may depend on specific surgical characteristics.

These associations may reflect shared perioperative mechanisms related to increased surgical complexity and extent of tissue manipulation. In patients with higher BMI, reduced surgical field visibility, greater tissue retraction, and higher intraoperative airway pressures may be associated with increased mechanical stress on laryngeal structures. Similarly, total thyroidectomy and larger thyroid volume require greater surgical exposure and dissection, including bilateral manipulation of the recurrent laryngeal nerves and greater traction, or occasional division, of the strap muscles. Longer duration of surgery further increases cumulative exposure to tissue manipulation and may be associated with reduced precision of dissection due to surgical fatigue. Previous research has shown that longer duration of thyroid surgery is associated with a higher risk of postoperative voice change and throat discomfort, particularly when procedures exceed approximately two hours [26]. In addition, enlarged thyroid volume has been associated with a longer duration of surgery, which may partly explain its association with later recovery stages. Dong et al. reported that thyroid volume was an independent risk factor for difficult thyroidectomy, defined as prolonged surgery duration, supporting a potential association between gland size, operative complexity, and postoperative objective voice recovery patterns [27].

Collectively, these factors may be associated with a more pronounced inflammatory response, postoperative soft tissue edema, and subclinical neural injury. Intraoperative traction, compression, or thermal exposure may result in transient neuropraxia or mild axonotmesis of the recurrent or external branch of the superior laryngeal nerve, even in the absence of macroscopically evident injury [28,29]. Such combined soft tissue effects and subtle neural dysfunction may impair fine neuromuscular control of phonation without causing clinically apparent impairment of vocal fold mobility, thereby contributing to a later recovery stage and persistent voice impairment in a subset of patients.

Furthermore, manipulation and potential division of the strap muscles may alter the integrity of the external laryngeal framework. As these muscles contribute to laryngotracheal positioning and movement during phonation, their disruption, together with postoperative scarring and adhesions between the laryngotracheal complex and surrounding tissues, may impair laryngeal mobility and pitch control [30,31]. This provides an additional, potential explanation for the observed association between more extensive surgical dissection and later recovery stages, consistent with previous reports associating more extensive thyroid resections with longer recovery of voice parameters [15,17].

These proposed mechanisms are speculative and should be interpreted only as potential explanations for the observed associations, while the exact mechanisms underlying postoperative voice alterations after thyroidectomy remain unknown, as they were not directly evaluated in the present study.

Significant interaction effects were observed between BMI and both duration and type of surgery. The interaction between BMI and duration of surgery indicates that the association between BMI and postoperative recovery differed according to operative duration, particularly when comparing patients recovering at the third postoperative assessment with those who had not recovered by the final assessment. Similarly, the significant BMI × type of surgery interaction suggests that the association between BMI and recovery patterns differed according to the surgical procedure. This effect was particularly evident for the comparison between recovery at the third postoperative assessment and absence of recovery by the final follow-up. In contrast, no significant interaction was observed between BMI and preoperative thyroid volume. These findings suggest that the influence of BMI on postoperative voice recovery may depend on specific aspects of surgical exposure and complexity rather than on thyroid size alone.

This study has several strengths. The relatively large cohort, prospective follow-up, and standardized multidimensional voice assessment enhance the reliability of the findings. Furthermore, exclusion of patients with confirmed vocal fold paresis or paralysis allowed analysis of functional voice recovery independent of evident RLN injury.

Several limitations should also be acknowledged.

Forty-seven patients were lost to follow-up before the first postoperative assessment, and reasons for this attrition were not systematically recorded. A formal comparison of baseline characteristics between these patients and those retained in the final analytic cohort was not performed in the present analysis. Consequently, the possibility of differential attrition and its potential impact on the representativeness of the study cohort cannot be excluded; systematic comparison of baseline characteristics between completers and non-completers is recommended in future prospective studies.

All procedures were performed by a single high-volume thyroid surgeon, which improved procedural consistency and reduced inter-surgeon variability. However, this may limit external validity and generalizability of the findings, as the observed recovery patterns may partially reflect operator-specific surgical technique and perioperative management.

Recovery was assessed only at predefined postoperative intervals rather than continuously, meaning that the exact timing of normalization between visits remains unknown. Consequently, recovery stage reflects interval-based recovery categories rather than true time-to-event data, limiting temporal precision and restricting interpretation of recovery dynamics. In addition, some recovery-stage subgroups were relatively small (e.g., stage 1, *n* = 43), and the correspondingly wide confidence intervals observed for some predictors indicate that the associated parameter estimates should be interpreted with caution.

In addition, patients who achieved complete normalization of all voice parameters were not reassessed at subsequent follow-up visits. For repeated-measures analyses, normalized values were carried forward to later time points under the assumption that recovery remained stable once achieved. Although this assumption was considered clinically reasonable given the definition of recovery used in the study, the possibility of unobserved fluctuations after recovery cannot be completely excluded.

The relatively small proportion of normal weight participants (14%) compared with overweight and obesity groups may have reduced the precision and stability of parameter estimates involving this subgroup. In addition, the BMI distribution of the present cohort may limit generalizability to populations with lower prevalence of overweight and obesity.

Furthermore, the study was conducted at a single tertiary center, which may limit generalizability, as outcomes may reflect the patient population and perioperative protocols specific to our institution.

All patients underwent general anesthesia with endotracheal intubation. While videostroboscopy at the first postoperative assessment confirmed vocal fold integrity, we cannot entirely exclude the potential influence of intubation on early postoperative voice changes. Use of a supraglottic airway device, such as an i-gel, may reduce this effect, but was not employed in this cohort [32].

Patient-reported voice outcomes, which represent an important dimension of voice-related quality of life [33], were not part of the predefined outcome framework for this analysis, which was specifically designed to examine the interaction between patient-related and surgical factors in objective postoperative voice parameter normalization following thyroidectomy. Accordingly, the findings reflect objective voice outcomes rather than patient-perceived vocal handicap. This distinction should be considered when interpreting the clinical implications, as previous studies have reported notable discrepancies between objective voice measures and patient-reported symptoms following thyroidectomy [34,35], consequently limiting the direct clinical applicability of objectively measured voice outcomes alone. Therefore, the present conclusions pertain primarily to objective voice recovery and should not be interpreted as direct indicators of subjective voice-related quality of life or patient-experienced functional vocal recovery. More broadly, statistically significant associations identified in this study do not necessarily equate to clinically meaningful impairment for every affected patient; the clinical significance of delayed objective voice normalization should be weighed alongside patient-reported vocal function and occupational voice demands, which were not captured in the present objective outcome framework.

Finally, the EBSLN was not routinely identified intraoperatively, in contrast to the RLN. Although standard surgical precautions were applied, the possibility of unrecognized macroscopic EBSLN injury (e.g., transection), in addition to already discussed microscopic injury related to traction or thermal exposure, cannot be entirely excluded. This is particularly relevant because even in cases of complete EBSLN transection, changes to the everyday speaking voice may be minimal but persistent, while laryngeal findings are often subtle and lack pathognomonic features that would allow precise diagnosis of EBSLN impairment [36,37]. Because EBSLN identification was not performed routinely and its status was not systematically documented, the potential influence of EBSLN injury on postoperative voice recovery could not be directly assessed. Therefore, unrecognized EBSLN injury cannot be entirely excluded and may represent a potential confounding factor in the interpretation of postoperative voice recovery patterns, particularly for objective voice parameters related to pitch control, while functional effects may be more pronounced during singing voice tasks. Similarly, intraoperative RLN-related factors that may potentially influence postoperative voice outcomes, such as anatomical variations or subjective assessments of nerve traction during dissection, were not systematically recorded and therefore could not be analyzed. Their potential contribution to postoperative voice recovery patterns cannot be completely excluded.

Although multiple patient-related and surgical variables were examined simultaneously, postoperative voice recovery is likely influenced by additional factors not included in the present analysis. Therefore, the observed associations should be interpreted within the context of the variables examined in the present study.

The findings of this study have several practical implications for the management of patients undergoing thyroidectomy. First, patient-related factors such as higher BMI and surgical factors including total thyroidectomy, longer duration of surgery, and enlarged thyroid volume should be considered when counseling patients regarding expected timelines for objective voice recovery. Patients with these risk factors may benefit from closer postoperative follow-up to monitor the course of objective voice recovery and could be considered for supportive interventions, such as voice therapy where clinically indicated [38], in addition to tailored perioperative strategies aimed at minimizing tissue trauma and facilitating postoperative recovery of objective voice parameters. Second, the observed interactions between BMI and surgical characteristics highlight the importance of considering patient-related and procedural factors jointly rather than in isolation. In particular, careful intraoperative planning and efforts to minimize operative duration where clinically feasible may be especially relevant in patients with higher BMI. Because BMI, thyroid volume, type of surgery, and duration of surgery are likely interrelated, and this study was observational, these associations should be interpreted as informing risk awareness and individualized counseling rather than as evidence that shortening operative duration would, in itself, causally improve voice recovery. Finally, these results support the potential utility of multidimensional voice assessment as part of routine perioperative evaluation, facilitating the recognition of patients who may experience delayed normalization of objective voice parameters. This may be particularly relevant for individuals with high vocal demands, such as professional voice users, in whom even subtle objective alterations in voice parameters may translate into clinically meaningful functional limitations. Collectively, these insights may inform risk-aware patient counseling and postoperative follow-up planning.

## 5. Conclusions

In this prospective cohort study, postoperative voice recovery stage was associated with BMI and several surgical characteristics, including type and duration of surgery and preoperative thyroid volume. Significant interactions were observed between BMI and duration of surgery and between BMI and type of surgery, suggesting that the association between BMI and postoperative recovery may depend on specific surgical characteristics. No significant interaction was observed between BMI and thyroid volume. Awareness of these associations may support postoperative risk stratification and more individualized monitoring strategies. However, these findings should be interpreted within the limitations of an observational single-center design and an objective outcome framework. Future studies should incorporate time-to-event analytical approaches and appropriate ordinal regression models to allow more precise characterization of postoperative voice recovery trajectories and individualized risk estimation. In addition, multicenter studies involving broader patient populations and varying surgical practices are warranted to improve external validity and assess reproducibility across different clinical settings. Integration of objective voice measures with patient-reported outcomes may further clarify the relationship between objectively measured voice recovery and patient-perceived vocal function.

## Figures and Tables

**Table 1:** Characteristics of the study cohort.

		*N*	%
Total		292	100
Recovery stage	1	43	14.7
	2	141	48.3
	3	44	15.1
	4	64	21.9
Sex	Male	46	15.8
	Female	246	84.2
Age	20–39	75	25.7
	40–59	165	56.5
	60–79	52	17.8
Body mass index	Normal weight	41	14.0
	Overweight	125	42.8
	Obesity	126	43.2
Type of surgery	Lobectomy	148	50.7
	Total thyroidectomy	144	49.3
Duration of surgery	≤90 min	155	53.1
	>90 min	137	46.9
Preoperative thyroid volume	Normal	135	46.2
	Enlarged	157	53.8

**Table 2:** Summary of multinomial logistic regression models examining interactions between BMI and clinical variables.

Interaction Term	Model χ^2^ (*df*)	*p*	Pearson GOF *p*	Nagelkerke *R*^2^	Interaction LR χ^2^ (*df*)	*p*
BMI × duration of surgery	94.70 (15)	<0.001	0.304	0.302	12.10 (3)	0.007
BMI × type of surgery	97.77 (15)	<0.001	0.738	0.31	15.17 (3)	0.002
BMI × thyroid volume	86.32 (15)	<0.001	0.372	0.279	3.72 (3)	0.294

Note. All models included BMI, duration of surgery, type of surgery, and thyroid volume, in addition to the specified interaction term. BMI and duration of surgery were mean-centered before calculation of interaction terms. Body mass index (BMI); goodness-of-fit (GOF); likelihood ratio (LR).

**Table 3:** Multinomial logistic regression estimates for the interaction between BMI and type of surgery.

Predictor	Stage 1 vs. 4 OR (95% CI)	*p*	Stage 2 vs. 4 OR (95% CI)	*p*	Stage 3 vs. 4 OR (95% CI)	*p*
BMI	1.000 (0.879–1.138)	0.998	0.999 (0.889–1.123)	0.989	0.902 (0.752–1.082)	0.268
Duration of surgery	0.984 (0.968–1.001)	0.063	0.983 (0.973–0.994)	0.002	0.985 (0.972–0.999)	0.041
BMI × surgery	0.723 (0.404–1.295)	0.275	0.945 (0.790–1.130)	0.534	1.362 (1.075–1.724)	0.010
Type of surgery	21.417 (2.842–161.384)	0.003	0.736 (0.318–1.702)	0.474	0.422 (0.127–1.408)	0.161
Thyroid volume	3.216 (1.349–7.664)	0.008	1.366 (0.730–2.556)	0.329	1.229 (0.543–2.784)	0.621

Note. Recovery stage 4 was the reference outcome category. BMI was mean-centered before calculating the interaction term.

**Table 4:** Multinomial logistic regression estimates for the interaction between BMI and duration of surgery.

Predictor	Stage 1 vs. 4 OR (95% CI)	*p*	Stage 2 vs. 4 OR (95% CI)	*p*	Stage 3 vs. 4 OR (95% CI)	*p*
BMI	1.050 (0.872–1.263)	0.607	0.961 (0.875–1.056)	0.409	1.133 (1.008–1.273)	0.036
Duration of surgery	0.984 (0.968–1.001)	0.060	0.983 (0.972–0.993)	0.001	0.979 (0.965–0.994)	0.005
BMI × duration	1.002 (0.997–1.006)	0.429	1.000 (0.997–1.002)	0.746	1.004 (1.001–1.007)	0.011
Type of surgery	18.572 (3.118–110.624)	0.001	0.716 (0.310–1.655)	0.435	0.347 (0.110–1.099)	0.072
Thyroid volume	3.298 (1.380–7.878)	0.007	1.363 (0.729–2.549)	0.333	1.340 (0.593–3.028)	0.482

Note. Recovery stage 4 was the reference outcome category. BMI was mean-centered before calculating the interaction term.

**Table 5:** Multinomial logistic regression estimates for the interaction between BMI and thyroid volume.

Predictor	Stage 1 vs. 4 OR (95% CI)	*p*	Stage 2 vs. 4 OR (95% CI)	*p*	Stage 3 vs. 4 OR (95% CI)	*p*
BMI	1.014 (0.855–1.203)	0.870	1.067 (0.927–1.229)	0.368	1.141 (0.964–1.351)	0.125
Duration of surgery	0.984 (0.968–1.000)	0.057	0.983 (0.973–0.994)	0.002	0.985 (0.971–0.998)	0.025
BMI × thyroid volume	1.004 (0.803–1.254)	0.973	0.876 (0.734–1.044)	0.140	0.939 (0.759–1.161)	0.560
Type of surgery	16.549 (2.930–93.467)	0.001	0.753 (0.325–1.745)	0.508	0.390 (0.130–1.174)	0.094
Thyroid volume	2.938 (1.209–7.139)	0.017	1.377 (0.734–2.583)	0.319	1.332 (0.584–3.034)	0.496

Note. Recovery stage 4 was the reference outcome category. BMI was mean-centered before calculating the interaction term. Reference categories for type of surgery and thyroid volume were total thyroidectomy and enlarged thyroid volume, respectively.

## Data Availability

The original contributions presented in this study are included in the article. Further inquiries can be directed to the corresponding author.

## References

[1] Ringel M.D., Sosa J.A., Baloch Z., Bischoff L., Bloom G., Brent G.A., Brock P.L., Chou R., Flavell R.R., Goldner W. (2025). 2025 American Thyroid Association Management Guidelines for Adult Patients with Differentiated Thyroid Cancer. Thyroid.

[2] Borel F., Christou N., Marret O., Mathonnet M., Caillard C., Bannani S., Drui D., Espitalier F., Blanchard C., Mirallié E. (2018). Long-term voice quality outcomes after total thyroidectomy: A prospective multicenter study. Surgery.

[3] Ling Y., Zhao J., Zhao Y., Li K., Wang Y., Kang H. (2020). Role of intraoperative neuromonitoring of recurrent laryngeal nerve in thyroid and parathyroid surgery. J. Int. Med. Res..

[4] Lang B.H., Wong C.K., Ma E.P. (2016). A systematic review and meta-analysis on acoustic voice parameters after uncomplicated thyroidectomy. Laryngoscope.

[5] Li C., Lopez B., Fligor S., Broekhuis J.M., Maeda A., Duncan S., Chen H.W., Choudhary A., Budwani S., Hasselgren P.-O. (2021). Long-term voice changes after thyroidectomy: Results from a validated survey. Surgery.

[6] Sung E.S., Kim K.Y., Yun B.R., Song C.M., Ji Y.B., Lee J.C., Tae K. (2018). Long-term functional voice outcomes after thyroidectomy, and effect of endotracheal intubation on voice. Eur. Arch. Otorhinolaryngol..

[7] Goswami S., Peipert B.J., Mongelli M.N., Kurumety S.K., Helenowski I.B., Yount S.E., Sturgeon C. (2019). Clinical factors associated with worse quality-of-life scores in United States thyroid cancer survivors. Surgery.

[8] Park J.O., Bae J.S., Lee S.H., Shim M., Hwang Y., Joo Y., Park Y.H., Sun D. (2016). The Long-Term Prognosis of Voice Pitch Change in Female Patients After Thyroid Surgery. World J. Surg..

[9] Tedla M., Chakrabarti S., Suchankova M., Weickert M.O. (2016). Voice outcomes after thyroidectomy without superior and recurrent laryngeal nerve injury: VoiSS questionnaire and GRBAS tool assessment. Eur. Arch. Otorhinolaryngol..

[10] D’haeseleer E., Huvenne W., Vermeersch H., Meerschman I., Imke K., Servayge L., Versavel O., Van Lierde K. (2021). Long-term voice quality outcome after thyroidectomy without laryngeal nerve injury: A prospective 10 year follow up study. J. Commun. Disord..

[11] Rosa M.S., Dell’Era V., Campagnoli M., Campini S., Barozza O., Borello G., Garzaro M., Valletti P.A. (2024). Vocal Outcome Following Thyroidectomy for Differentiated Thyroid Carcinoma. J. Clin. Med..

[12] Sahli Z., Canner J.K., Najjar O., Schneider E.B., Prescott J.D., Russell J.O., Tufano R.P., Zeiger M.A., Mathur A. (2019). Association Between Age and Patient-Reported Changes in Voice and Swallowing After Thyroidectomy. Laryngoscope.

[13] Jain P.V., Roy D., Manikantan K., Sharan R., Tshering P., Arun P. (2021). Contribution of Weight and Volume of the Extirpated Thyroid Gland on Voice Alterations After Total Thyroidectomy in Patients With Papillary Carcinoma of the Thyroid. J. Voice.

[14] Vicente D.A., Solomon N.P., Avital I., Henry L.R., Howard R.S., Helou L.B., Coppit G.L., Shriver C.D., Buckenmaier C.C., Libutti S.K. (2014). Voice outcomes after total thyroidectomy, partial thyroidectomy, or non-neck surgery using a prospective multifactorial assessment. J. Am. Coll. Surg..

[15] Kim G.J., Bang J., Shin H.I., Kim S.-Y., Bae J.S., Kim K., Kim J.S., Hwang Y.-S., Shim M.-R., Sun D.-I. (2023). Persistent subjective voice symptoms for two years after thyroidectomy. Am. J. Otolaryngol..

[16] Kim C.S., Park J.O., Bae J.S., Lee S., Joo Y., Park Y., Hwang Y., Shim M., Sun D. (2018). Long-Lasting Voice-Related Symptoms in Patients Without Vocal Cord Palsy After Thyroidectomy. World J. Surg..

[17] Nam I.C., Bae J.S., Lee S.H., Kim J., Hwang Y., Shim M., Kim G., Park J., Park Y., Sun D. (2023). Prospective voice assessment after uncomplicated thyroidectomy: A comprehensive analysis of a single centre experience. Clin. Otolaryngol..

[18] Šimić Prgomet I., Frkanec S., Gugić Radojković I., Prgomet D. (2024). Prospective Voice Assessment After Thyroidectomy Without Recurrent Laryngeal Nerve Injury. Diagnostics.

[19] von Elm E., Altman D.G., Egger M., Pocock S.J., Gøtzsche P.C., Vandenbroucke J.P., STROBE Initiative (2008). The Strengthening the Reporting of Observational Studies in Epidemiology (STROBE) statement: Guidelines for reporting observational studies. J. Clin. Epidemiol..

[20] Hirano M., Arnold G.E., Winckel F., Wyke B.D. (1981). Psycho-acoustic evaluation of voice. Clinical Examination of Voice.

[21] WEVOSYS Medical Technology GmbH (2017). Voice Protocol Norms [Software Manual]. Baunach (DE): WEVOSYS Medical Technology GmbH. https://mmsp.com.au/mmsp/wp-content/uploads/2019/08/lingWAVES_Voice_Protocol_Norms_2017_09_25.pdf.

[22] World Health Organization (2000). Obesity: Preventing and Managing the Global Epidemic: Report of a WHO Consultation.

[23] Hegedüs L., Perrild H., Poulsen L.R., Andersen J.R., Holm B., Schnohr P., Jensen G., Hansen J.M. (1983). The determination of thyroid volume by ultrasound and its relationship to body weight, age, and sex in normal subjects. J. Clin. Endocrinol. Metab..

[24] Belafsky P.C., Postma G.N., Koufman J.A. (2002). Validity and reliability of the reflux symptom index (RSI). J. Voice.

[25] Belafsky P.C., Postma G.N., Koufman J.A. (2001). The validity and reliability of the reflux finding score (RFS). Laryngoscope.

[26] Chawaka H.J., Teshome Z.B. (2023). The Underreported Postoperative Suffering after Thyroid Surgery: Dysphagia, Dysphonia, and Neck Pain-A Cross-Sectional Study. Anesthesiol. Res. Pract..

[27] Dong M., Song J.L., Hu L.L., Hong C.-C., Nie X.-Y., Wang Z., Liao S.-C., Yao F. (2023). Analysis of preoperative influential factors and construction of a predictive nomogram of difficult thyroidectomy. BMC Surg..

[28] Kim S.Y., Kim G.J., Lee D.H., Bae J.-S., Lee S.-H., Kim J.-S., Hwang Y.-S., Shim M.-R., Park Y.-H., Sun D.-I. (2020). Voice change after thyroidectomy without vocal cord paralysis: Analysis of 2,297 thyroidectomy patients. Surgery.

[29] Liu M.Y., Chang C.P., Hung C.L., Huang S. (2020). Traction Injury of Recurrent Laryngeal Nerve During Thyroidectomy. World J. Surg..

[30] Lee J.S., Kim J.P., Ryu J.S., Woo S.H. (2018). Effect of wound massage on neck discomfort and voice changes after thyroidectomy. Surgery.

[31] Zhang B., Lv B., Liu N. (2021). Anterolateral approach for thyroid swellings: Impact on postoperative voice outcomes. Gland Surg..

[32] Ning M., Zhong W., Li J., Wang T., Lu Y. (2022). Comparison between I-gel^®^ and endotracheal intubation in terms of the incidence of postoperative sore throat following thyroid surgery: A randomized observational trial. Am. J. Transl. Res..

[33] Borel F., Tresallet C., Hamy A., Mathonnet M., Lifante J.-C., Brunaud L., Marret O., Caillard C., Espitalier F., Drui D. (2020). Self-assessment of voice outcomes after total thyroidectomy using the Voice Handicap Index questionnaire: Results of a prospective multicenter study. Surgery.

[34] Huang T.Y., Yu W.V., Chiang F.Y., Wu C.-W., Fu S.-C., Tai A.-S., Lin Y.-C., Tseng H.-Y., Lee K.-W., Lin S.-H. (2021). Correlation Between Objective and Subjective High-Pitched Voice Impairment in Patients After Thyroid Surgery. Front. Endocrinol..

[35] Chrut A., Okła S., Koziel D., Kubala-Kukuś A., Gluszek S. (2026). Acoustic analysis and self-assessment of changes in the voice after thyroidectomy. Otolaryngol. Pol..

[36] Le Pape G., Lazard D.S., Gatignol P., Tresallet C., Pillot-Loiseau C. (2021). Voice modulation, self-perception and motor branch of the superior laryngeal nerve. Eur. Ann. Otorhinolaryngol. Head Neck Dis..

[37] Potenza A.S., Araujo Filho V.J.F., Cernea C.R. (2017). Injury of the external branch of the superior laryngeal nerve in thyroid surgery. Gland Surg..

[38] Nam I.C., Bae J.S., Chae B.J., Shim M.R., Hwang Y.S., Sun D.I. (2013). Therapeutic approach to patients with a lower-pitched voice after thyroidectomy. World J. Surg..

